# Branching of Titanium Nanorods

**DOI:** 10.3390/nano11051070

**Published:** 2021-04-22

**Authors:** Nosirudeen Abayomi Yussuf, Hanchen Huang

**Affiliations:** 1 Department of Mechanical and Industrial Engineering, Northeastern University, Boston, MA 02115, USA; yussuf.a@northeastern.edu; 2 Department of Mechanical Engineering, University of North Texas, Denton, TX 76203, USA

**Keywords:** nanorod, glancing angle deposition (GLAD), branching, titanium, hexagonal close-packed (HCP)

## Abstract

One dimensional titanium nanorod structures formed by glancing angle physical vapor deposition have branches while other hexagonal closed packed metals do not. Based on physical vapor deposition and characterizations using electron microscopy and X-ray diffraction, this paper reports that Ti nanorod branching occurs at a low homologous temperature of 0.28. The side surface of the nanorods consists of {$10\bar{1}1$} facets arranged in a zigzag shape. Further, branches form on the {$10\bar{1}1$} side facets that are parallel to the deposition flux. The length of the branches increases as they are farther away from the nanorod top and tend to reach a constant. The top surface facet of Ti nanorods is {0001} and that of the branches is {$10\bar{1}1$}. The insight into conditions for branching, together with the determination of the morphology and crystal orientation of the branches, lay the foundation for further studies of branching mechanisms and driving force.

## 1. Introduction

It is common to grow one dimensional nanostructures using physical vapor deposition under glancing angle deposition (GLAD) conditions by taking advantage of geometrical shadowing [1,2]. These nanostructures take the forms of rods, springs, zigzags, and blades [3,4,5,6,7,8,9]. A key factor that makes these structures nano-sized is the limited surface diffusion that is dictated by the three-dimensional (3D) Ehrlich–Schwoebel (ES) barrier [10,11,12]. Based on this concept of the 3D ES barrier, we have developed a closed form theory to predict the diameter of nanorods [13,14]; an accompanying theory also provides the prediction of nanorod separation. Guided by these theories, we have minimized the diameter and maximized the separation to experimentally realize the smallest and well-separated metallic nanorods of Cu, Ag, and Au [15]. 

Variation of one-dimensional structures by GLAD is possible through the control of processing parameters, including surface diffusion, geometrical shadowing, and intrinsic crystal properties like stacking faults and impurity concentration among others. For example, as a result of the rotation of the incident flux around a patterned substrate, branching in the cubic structure has been achieved as stacking faults form on Cu {111} surfaces [9] or with insufficient atomic diffusion for refractory metal Ta [16]. These branches typically follow the same crystal structure with the nanorod when they nucleate and grow, but they may also form grain boundaries with the nanorod.

The majority of the GLAD literature focuses on face-centered-cubic (FCC), body-centered-cubic (BCC) and their compounds [7,9,16,17,18,19,20,21,22]. In contrast, hexagonal close-packed (HCP) metals and their compounds have not received as much attention despite the many beneficial properties they possess. In comparison, for both HCP and cubic metals, close-packed planes dominate the growing surface of nanorods. For FCC, the {111} surfaces of nanorods tend to face the incoming flux [23,24]. For BCC, the {110} or {112} surfaces of the nanorods tend to face the incoming flux [25]. For HCP, the {0001} surfaces tend to face the incoming flux [26,27,28,29]. The formation of these surfaces is the result of minimizing surface energy and maximizing surface diffusion.

HCP crystal structures are anisotropic, and surface diffusion is also anisotropic [28,29,30]. As a result, HCP nanorods generally are anisotropic, with a width to thickness ratio being substantially different from 1:1, when the incidence angle of the deposition flux is fixed. However, it is possible to decrease the aspect ratio from as high as 10:1 to close to 1:1 by increasing the rate of substrate rotation [25,26,27]. As the substrate temperature increases, the anisotropy also decreases [20,31]. Among all HCP metals, only Ti nanorods have been reported so far to form branches [32].

The question is why Ti nanorods form branches while other HCP metals do not. To answer this question, we must ask more fundamental questions: (1) what the morphology and crystal orientations of the Ti nanorods and branches are, and (2) what deposition conditions lead to the branching. This paper reports experimental characterizations and analyses that aim to answer these two questions. Section 2 presents experimental methods, Section 3 presents experimental results and analyses, and Section 4 presents the conclusions.

## 2. Experimental Methods 

**Figure 1:**
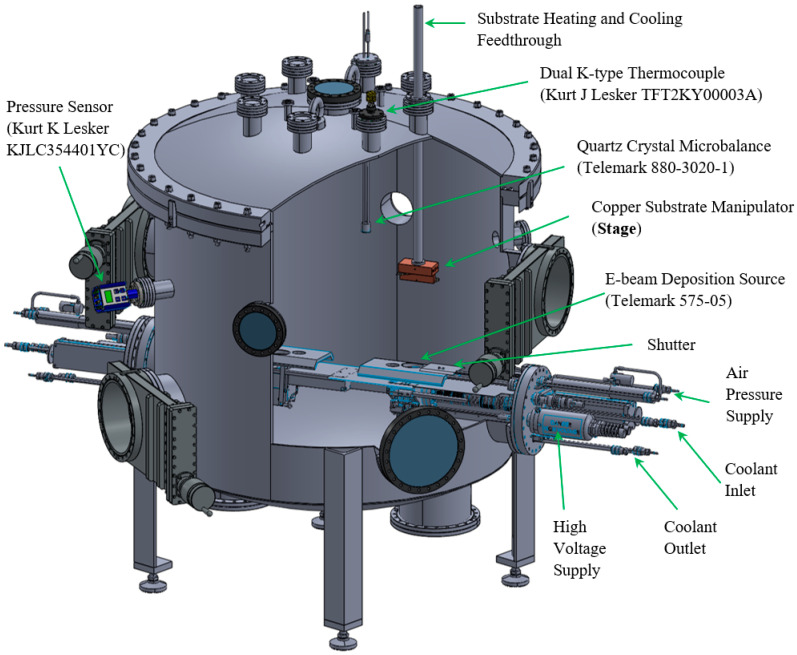
Schematic of the deposition chamber highlighting various components.

Ti nanorods are deposited on Si{001} substrates by using physical vapor deposition under GLAD conditions. The deposition chamber, as schematically shown in Figure 1, is an ultrahigh vacuum with a base pressure of 10^−4^ Pa and a working pressure of about 10^−5^ Pa during deposition. This working pressure is maintained using a turbomolecular pump. The deposition chamber is 1020 mm in diameter and features a source at the bottom and a copper substrate manipulator (stage) located 360 mm above it. The deposition source is from electron beam (e-beam) evaporation of Ti pellets (purity 99.995%, 6.35 mm diameter × 6.35 mm length). The stage is designed to hold a substrate of up to 1900 mm^2^ with an incidence angle range of 85–89° to the flux, and is fastened to a feedthrough for temperature control. The substrate is kept stationary during the entire deposition process with no azimuthal rotations. A temperature range of 103K–543K is achieved through substrate heating and cooling. A cartridge heater in the feedthrough enables the heating. Liquid nitrogen that is poured into the feedthrough enables the cooling. For each experiment, the desired temperature is held for more than three hours before deposition, and it is maintained using a Watlow Dual temperature controller. A K-type thermocouple is attached to the stage to monitor the temperature of the stage. The nominal deposition rate ranging from 0.05 nm/s to 2 nm/s is measured and read through a quartz crystal microbalance (QCM) located normal to the flux and adjacent to the substrate.

The Si{001} substrates used are ultrasonically cleaned sequentially in a bath of acetone, isopropyl alcohol and deionized water for 30 mins each and are then set to dry in atmospheric air. The cleaned Si{001} substrates are attached to the stage set at a glancing angle of 87° with the direction of the incident flux. The nominal deposition rate is set to 0.5 nm/s. This rate is achieved with a voltage of 10 kV and an emission current ranging from 70–110 mA. The temperature of the substrate is increased by 3K during deposition. The deposition time is 50 mins, corresponding to a nominal film thickness (with no porosity) of 1500 nm.

Nanorod morphology and microstructural analysis is performed using a high-resolution field scanning electron microscope (Hitachi S-4800, Tokyo, Japan). Under the accelerating voltage of 3 kV and with a working distance of 8 mm, the spatial resolution is 2 nm. The structure is characterized using a Cs-corrected transmission electron microscope (Thermo Fisher, TEM/STEM, FEI Titan Themis 300, Waltham, MA, USA). Under 300 kV, the spatial resolution reaches 0.07 nm and a diffraction detection diameter of 200 nm. Texture analysis is performed using X-ray diffraction (XRD, CuKa radiation of wavelength 0.154 nm, 40 KV, 44 mA, Rigaku ultima IV, Tokyo, Japan) for a sample size of 900 mm^2^ in area and 0.38 mm in total thickness of the Ti and the Si substrate. The nanorod dimensions are analyzed, measured and processed using the ImageJ Processing Program [33,34]. Angular dimensions are measured relative to the substrate normal. To prevent inconsistencies during the angular measurements of individual nanorods, twenty nanorods are selected for each measured value, and two branches are included when they are involved. The normal vector of the nanorod top surface and axial direction of the nanorod are measured relative to the substrate normal. The difference between the top surface facet of the nanorod and the top surface facet of the branches is measured directly, and so is the difference between the axial direction of the nanorod and that of the branches. The angles between the normal vector of the top surface facet of the nanorod and that of the top surface facet of the branches, as well as the angles formed by the side facets of the nanorods, are measured in two steps. First, the sample is rotated so that the normal vector of the top surface facet of the nanorod is in the viewing plane. Next, the sample is rotated around the normal vector of the nanorod top surface facet to maximize the angle. For each group of samples, the standard deviation is calculated to represent the error bar/uncertainty interval for the measurements.

## 3. Results and Analyses

**Figure 2:**
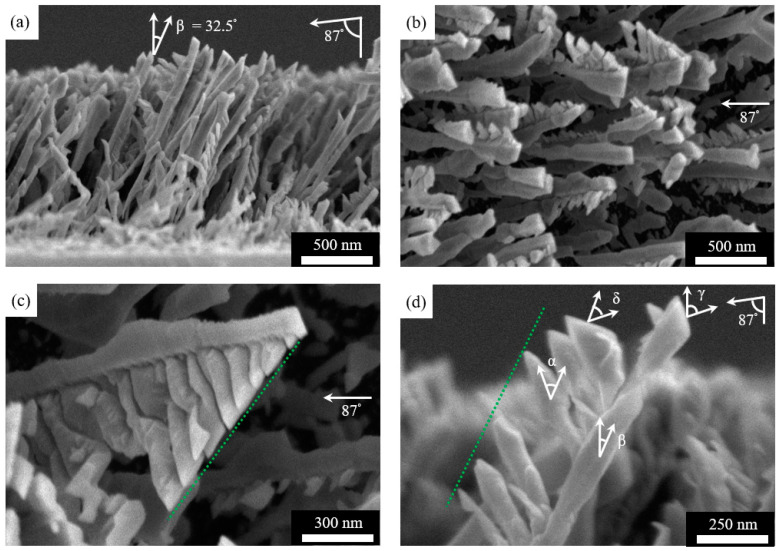
SEM images of Ti nanorods of (**a**) cross-section view and (**b**) top view, with the incidence angle of 87° and nanorod tilt angle of 32.5° shown. (**c**) Nanorod with the dotted line highlighting the increasing branch length. (**d**) Nanorod showing various angles, with the dotted line highlighting the constant length far away from the nanorod tip.

The first set of results in Figure 2 are SEM images of the Ti nanorods. These nanorods typically are 150–300 nm in diameter and 2400–2800 nm in length, and they are faceted. The cross-section view of Figure 2a shows that the nanorods tilt towards the flux direction, similar to nanorods of Al [35], Ag [36], Si, Ge, and Mo [37] deposited under similar conditions. When the incidence angle of the deposition flux is 87°, the tilt angle β measured relative to the substrate normal is 32.5 ± 3.5°. As described in Section 2, the averaging is over 20 nanorods, and the uncertainty of 3.5° is the standard deviation. The correlation of these two angles is only qualitatively in agreement with Tait’s cosine rule derived from geometric principles [38] or the empirical tangent rule [39], as expected. Beyond the generic features of morphology, Figure 2b shows the top view of the Ti nanorods. In particular, some nanorods have branches and others do not.

To determine conditions that lead to branching, we examine the SEM images. According to this examination, branching occurs on the side surfaces of the nanorods that are aligned parallel or close to being parallel with the incidence flux or contain the flux lines. The branches on a given side surface also have variable lengths. One feature is generally consistent—the branch length increases as it is further away from the nanorod top before reaching a constant length (Figure 2c). Figure 2d shows that branches tend to reach a constant length as they are far away from the nanorod top. However, this is not always the case as some of the nanorods in Figure 2b reveal, presumably due to changing shadowing conditions during growth.

**Figure 3:**
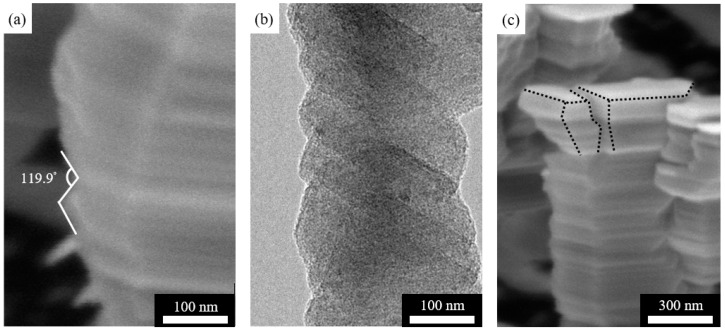
(**a**) SEM image of a nanorod with faceted side surfaces, (**b**) TEM image of a nanorod with faceted side surfaces, and (**c**) SEM image of a bifurcated nanorod with no branches.

In an effort to understand where branches form, we note that the side surfaces of nanorods are faceted. Figure 3a shows an SEM image of the side facets, with the sample tilted so that the normal vector of the top surface of the nanorod is in the viewing plane and then rotated to maximize the angle at 119.9 ± 1.1°. Figure 3b shows a projection TEM image of the side facets to more clearly illustrate the faceting nature of the side surfaces; the angle in the TEM image is not maximized and therefore smaller than 119.9 ± 1.1°. The combination of Figure 3a,b establishes that side surfaces of Ti nanorods are faceted and the nearby facets form an angle of 119.9 ± 1.1°. In passing, we note that when branching does not happen, the nanorod bifurcates as the top surface becomes sufficiently large (Figure 3c).

**Figure 4:**
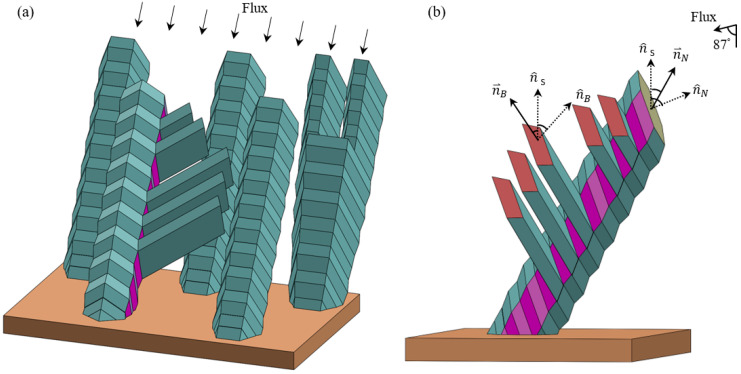
(**a**) Schematic of nanorods, including one with branches, one with bifurcation, and three with no bifurcation or branch; and (**b**) expanded view of the nanorod with branches, with relevant angles of Figure 2 marked, where ${\hat{n}}_{B}$, ${\hat{n}}_{S}$, ${\hat{n}}_{N}$ are the surface normal directions of the branch top, substrate and nanorod top, respectively while ${\vec{n}}_{B}$, ${\vec{n}}_{N}$ are the axial direction of the branch and nanorod.

Based on the observed morphologies of nanorods and their branches, we draw a schematic in Figure 4. In doing so, we take the Wulff construction of HCP Ti as reference for probable surface facets. The thermodynamically preferred {0001} surface is surrounded by six {$10\bar{1}1$} surfaces, and the next thermodynamically preferred surface is beyond {0001}. Indeed, prior experiments have reported the top surface of the nanorods as typically {0001} [27,29,40]. Our XRD and TEM characterizations—as presented later—also confirm that this is the case. We further assume that each Ti nanorod, together with its branches, is a single crystal; this is true as TEM and XRD experiments show later. Based on the 119.9 ± 1.1° angle observed in Figure 3a, we take that each side of the nanorod is covered by two complementary {$10\bar{1}1$} surfaces. In comparison with the theoretical value of 122.7°, our measured value of 119.9 ± 1.1° is expected to be slightly smaller since the measurements do not always correspond to the maximum angle that we aim for. We further assume that the top surface of the branches is {$10\bar{1}1$}, and this assumption is valid as the angle measurements show later.

Going beyond morphology, we quantify the relative orientations of nanorods and branches. The angle δ between these two surface facets ${\hat{n}}_{B}$ and ${\hat{n}}_{N}$ is measured to be 59.6 ± 1.1°, averaged over 20 nanorods and two branches for each nanorod. The theoretical value of the angle between {$10\bar{1}1$} and {0001} is 61.4°, which corresponds to the maximum angle of all experimental measurements. The agreement between 59.6 ± 1.1° and 61.4° confirms that (1) a nanorod and its branches form a single crystal and (2) the top facet of the branches is indeed {$10\bar{1}1$} if the top facet of the nanorods is {0001}, as shown in Figure 4.

Interestingly, the angle α between the axial direction of the branches ${\vec{n}}_{B}\;$and axial direction of the nanorods ${\vec{n}}_{N}$ varies over a wide range of 25.7–60.4°. Our SEM observation reveals that the side surfaces on which branches form align with the incident flux, and the alignment varies over a small range of angle. As the crystal orientation varies from one nanorod to another, the normal vector of the nanorod top surface facet ${\hat{n}}_{N}$ changes. Consequently, the side surface orientation changes relative to the flux. As a result of this and geometrical shadowing effects, the axial direction of the branches ${\vec{n}}_{B}$ varies over a wide range. This variation leads to the wide range of 25.7–60.4°.

It should be noted that nanorods are in three dimensions, and SEM images such as that of Figure 2d represent a two-dimensional projection. That is, angles measured in three dimensions are projected to a plane that contains the nanorod and the incidence flux in Figure 2d. In our results, the angles are those measured in three dimensions. The conversion from an angle measured in three dimensions to its projection on a particular plane is possible through a Stereographic projection chart [41,42]. For example, projection of angle γ is the same 71.4° (Figure 2d). However, for angle δ, the projection appears to be a smaller value of 45.9° (Figure 2d).

**Figure 5:**
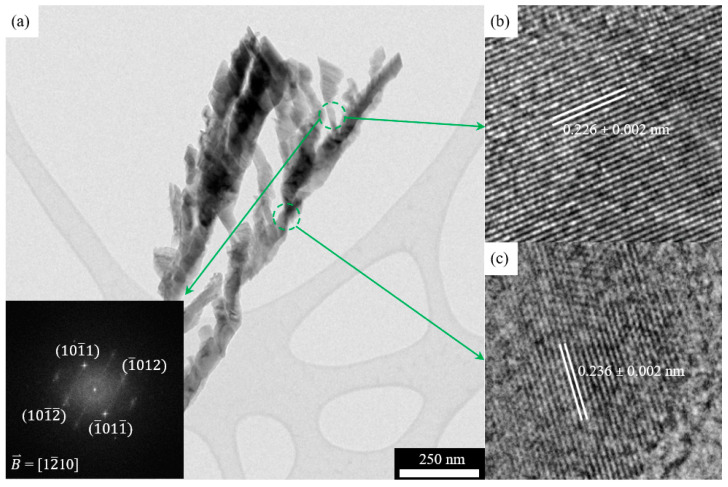
(**a**) HRTEM image of a Ti nanorod having branches with the inset showing selected area diffraction (SAED) pattern of the circled spot, (**b**) lattice spacing of the circled spot of the branch. (**c**) Lattice spacing of the circled spot of the nanorod.

To further confirm the single crystal nature of a nanorod and its branches, Figure 5 shows a high-resolution TEM characterization. The spacing of lattice planes parallel to the top surface facet of the nanorod is 0.236 nm, confirming that the top surface is {0001}. Further, the spacing of lattice planes parallel to the top surface facet of the nanorod branch is 0.226 nm. Together with the diffraction pattern in the inset of Figure 5a, it confirms that the top surface facet is {$10\bar{1}1$}, the same as the side facets.

**Figure 6:**
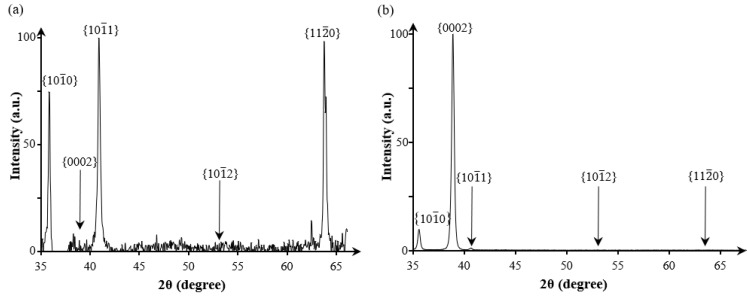
(**a**) XRD intensity as a function of angle 2θ for (**a**) Ti nanorods, (**b**) Ti polycrystalline thin film deposited with incidence angle of 0° and substrate temperature of 523K, and the other conditions are the same as for the nanorods.

Going beyond a single nanorod and its branches, we have also characterized the crystal orientations of the nanorods using XRD (Figure 6). Atomic planes parallel to the substrate are detectable by XRD [43]. We have taken the nanorod top surface facet as {0001} and the normal vector of this surface forms an angle γ of 54.2–88.5° with the substrate normal. However, the in-plane (relative to the top surface facet of the nanorod) texture is random. As a result, the conventional peak of {0001} should not be visible since it is 54.2–88.5° away from being parallel to the substrate. The next closed-packed planes {$10\bar{1}1$} can be parallel to the substate within 0°–30°, depending on the in-plane texture (relative to the top surface plane of the nanorod). Similarly, the {$11\bar{2}0$} can be parallel to the substate within 0°–60° and {$10\bar{1}0$} within 0°–30°. All the three peaks exist in Figure 6, as expected. Indeed, Figure 6a shows that the {0002} peak (corresponding to {0001} planes) is absent, in sharp contrast to the reference XRD for a randomly oriented polycrystalline Ti in Figure 6b.

Before concluding, we briefly discuss the effects of deposition conditions. When the substrate temperature is below 373K (θ ≃ 0.19), we do not observe branches of Ti nanorods. Instead, we observe nanorods with a width to thickness ratio as high as 8:1. However, within 373K to 423K (0.19 ≤ θ ≤ 0.22), branches form on some nanorods while the majority of the nanorods still have high aspect ratios. As the substrate temperature increases beyond 423K to 543K (0.22 ≤ θ ≤ 0.28), many nanorods have branches and few nanorods have high aspect ratios. Ideally, it would be interesting to see what happens when the homologous temperature goes above θ > 0.28. However, θ = 0.28 is the highest homologous temperature attainable in our system. In addition, there is no branch formation when the substrate rotation is increased to 1 rpm. In reference to the structural zone model for GLAD films [44], Ti nanorods form branches beyond Zone 1 (θ = 0.20).

## 4. Conclusions

We have used physical vapor deposition under GLAD conditions to grow Ti nanorods with branches, and analyzed them using SEM, TEM, and XRD techniques. Based on these analyses, we make six conclusions.

One, branches of Ti nanorods form when the substrate temperature reaches 543K (or 0.28 homologous temperature) and there is a fixed incidence angle of 87°. We note that as substrate temperature goes below 373K (or <0.19 homologous temperature), Ti nanorods with large aspect ratios are observed instead and branches do not form. In addition, branches do not form with fast substrate rotation.

Two, the side surface of Ti nanorods is in the form of zigzag morphology consisting of {$10\bar{1}1$} facets. For completeness, the top surface of the Ti nanorods is {0001} as previously reported [32].

Three, branches form on the {$10\bar{1}1$} facets of the nanorod that are parallel—or close to being parallel—to the deposition flux. No branches form on {$10\bar{1}1$} side surface facets that are far away from being parallel to the deposition flux.

Four, the top surface facet of the nanorod branches is {$10\bar{1}1$}, which forms an angle of 59.6 ± 1.1° with the top surface facet of the nanorod {0001}.

Five, the angle between the axial direction of a nanorod and that of its branches varies in a wide range of 25.7–60.4°, as the crystal orientation of the nanorod varies.

Six, the length of the nanorod branches increases as they are farther away from the nanorod top and tend to reach a constant length. However, some do not reach a constant presumably due to a changing shadowing environment.

## Data Availability

Not Applicable.
